# Effects of the Type of Sports Practice on the Executive Functions of Schoolchildren

**DOI:** 10.3390/ijerph19073886

**Published:** 2022-03-24

**Authors:** Falonn Contreras-Osorio, Iris Paola Guzmán-Guzmán, Enrique Cerda-Vega, Luis Chirosa-Ríos, Rodrigo Ramírez-Campillo, Christian Campos-Jara

**Affiliations:** 1 Department of Physical Education and Sports, Faculty of Sport Sciences, University of Granada, 18011 Granada, Spain; falonn.contreras@unab.cl (F.C.-O.); lchirosa@ugr.es (L.C.-R.); 2 Exercise and Rehabilitation Sciences Laboratory, School of Physical Therapy, Faculty of Rehabilitation Sciences, Universidad Andres Bello, Santiago de Chile 7591538, Chile; rodrigo.ramirez@unab.cl; 3 Faculty of Chemical-Biological Sciences, Universidad Autónoma de Guerrero, Chilpancingo de los Bravo 39087, Mexico; pao_nkiller@yahoo.com.mx; 4 Pedagogy in Physical Education and Health Career, Department of Health Science, Faculty of Medicine, Pontificia Universidad Católica de Chile, Santiago de Chile 7820436, Chile; ecerdav@uc.cl

**Keywords:** sport, physical activity and sport in youth, executive functions, physical fitness, human physical conditioning, muscle strength, musculoskeletal and neural physiological phenomena, physical education and training

## Abstract

There is a close relationship between the development of complex motor skills and executive functions during childhood. This study aimed to analyze the differences in different dimensions of executive functions in children practicing an open-skill sport (handball) and a closed-skill sport (athletics) and controls who did not participate in sports activities after a 12-week intervention period. School-aged male and female subjects (*n* = 90; mean ± standard deviation = 11.45 ± 0.68 years) participated in a non-randomized controlled study. Data analysis was performed using the STATA V.15 statistical software. The athletics intervention promoted semantic fluency (*p* = 0.007), whereas handball increased inhibition (*p* = 0.034). Additionally, physical activity improved in both intervention groups (*p* = < 0.001), whereas sprint performance improved in the handball group following intervention (*p* = 0.008), lower body muscular power improved in athletics (*p* = 0.04), and evidence of improvement in upper body muscular strength was noted in handball (*p* = 0.037). In turn, an increase in the Physical Activity Questionnaire for older Children score showed an association with the Standard Ten scores of executive functions. In conclusion, compared to controls, both athletics and handball induced meaningful improvements in physical activity and executive functions. However, sport-specific adaptations were noted after athletics (i.e., semantic fluency and lower body muscular power) and handball (i.e., inhibition, sprint, and upper-body muscular strength).

## 1. Introduction

Childhood is a relevant period for the development of complex motor skills and higher-order cognitive skills, such as executive functions [1,2,3,4], which allow for the regulation of thoughts and actions during goal-directed behavior [5]. There is consensus for the classification of these skills into three main dimensions: inhibition or inhibitory control, working memory, and cognitive flexibility [6,7], along with another set of higher-order processes, including reasoning, problem solving, and planning [8]. Current theoretical perspectives suggest that when interacting with their environment, children face situations that require control, coordination, and the integration of multiple body movements into a coherent and organized system, which drives cognitive development and enables the acquisition of new, varied, and complex motor skills that appear, for example, when learning a sport [2,9,10,11]. However, practicing motor tasks leads to automatization, at which point these cognitive resources, represented by executive functions and others (such as attention), can be allocated to new motor learning that requires the involvement of these skills [2,3]. This functional relationship is supported by studies demonstrating the parallel activation of brain regions, such as the prefrontal cortex, cerebellum, and basal ganglia, involved in the performance of complex motor tasks and executive functions [4,12,13]. 

The executive function of children and adolescents is associated with physical activity [14,15,16,17,18], particularly with sports practice, which brings together a set of characteristics compatible with the requirements necessary to promote its development [19,20,21,22]. This includes a combination of physical exercise and cognitively demanding activities that progressively become more challenging depending on the structure of each sport, and at the same time, these factors are linked to the interests of those who practice them, which favors motivation [23,24,25,26]. This relationship is especially true in the case of open-skill sports (e.g., handball), where changing and relatively unpredictable environmental situations require anticipating and making decisions in consideration of multiple variables in a dynamic and flexible manner [27,28,29]. In addition to being engaging, cognitively challenging, and goal-focused, sports foster children’s emotional and social development [11,24,30,31], boosting personal commitment, confidence, and pride in achieving expected accomplishments [20,21,22,23,27,32,33]. Recently, a meta-analysis demonstrated that sports programs for healthy children and adolescents have a significant effect on the executive function of the participants (working memory ES −1.25 [95% CI −1.70 to −0.79], *p* = 0.001; inhibitory control ES −1.30 [95% CI −1.98 to −0.63], *p* < 0.00001; cognitive flexibility ES −1.52 [95% CI −2.20 to −0.83], *p* < 0.00001) [34].

Studies analyzing the relationship between different types of sport and executive functions have mainly focused on the adult population [21,35,36,37,38,39]. This evidence suggests that athletes outperform their non-athlete peers in different executive function dimensions and that different types of sporting experiences are associated with better performance in specific domains of executive functions [35,40]. It also proposes that the development of executive functions might be favored to a greater extent by the practice of open-skill sports [21,35,36,39]. In the case of children, cross-sectional studies comparing different types of sports also support the idea that those who participate in open-skill sports (basketball, volleyball, soccer, korfball, hockey, and martial arts) demonstrate superior executive function performance in comparison with their peers who engage in closed-skill sports (rhythmic gymnastics, cycling, swimming, classical ballet, and athletics) and non-athlete controls [28,41]. Longitudinal studies, such as those by Alesi et al. [32], Ishihara et al. [42], and Schmidt et al. [33], highlight the importance of practicing open-skill sports (soccer, tennis, floorball, and basketball) to promote the development of executive functions in children, demonstrating significant improvements in working memory and cognitive flexibility. However, the specific associations between each sport and the main executive function dimensions have been poorly addressed [33,34].

Based on the evidence that shows the positive effect of sports practice on executive functions [34] and using previous studies that have analyzed the differences in the executive function of children practicing different types of sports as a reference [28,41,43], the study aimed to analyze the differences in different dimensions of executive function in children practicing an open-skill sport (handball) and a closed-skill sport (athletics) and controls who did not participate in sports activities after a 12-week intervention period. Based on previous studies [28,41,44,45], we hypothesized that handball and athletics practice would have an effect on different domains of executive functions, such as working memory, cognitive flexibility, inhibition, and planning. We further hypothesized that positive changes in executive function would be associated with improvements in participants’ daily physical activity levels and physical fitness (i.e., aerobic fitness, sprint performance, and lower and upper body muscular power and strength, respectively).

Therefore, intervention programs were developed within the school context, through extracurricular handball and athletics programs, in addition to regular physical education sessions. The control group was only exposed to regular physical education sessions. The present study provides relevant information for practitioners regarding the effects of different sport modalities on executive functions in schoolchildren, highlighting the important role of sport in enhancing the cognitive performance of students, in addition to its physical fitness benefits.

## 2. Materials and Methods

### 2.1. Design and Participants

We developed a longitudinal study with pre- and post-test measures, with an intervention period of 12 weeks, which is an adequate sport-based intervention period to improve executive function in school-age participants [34]. Boys (*n* = 46) and girls (*n* = 44), aged 10–12 years (mean ± standard deviation = 11.45 ± 0.68 years), of a high socioeconomic status participated in this study [46]. The students were classified into three groups: (1) non-athletes: boys and girls who did not participate in sports in addition to their physical education class at school, (2) boys and girls who participated in an extracurricular handball workshop, and (3) boys and girls who participated in an extracurricular athletics workshop (track events only, e.g., 400 m). The study protocol was approved by the ethical review board from the Scientific Ethics Committee of the Universidad Andres Bello, Chile (N° 009/2021). The procedure was in accordance with the latest version of the Declaration of Helsinki (2013).

The exclusion criteria were as follows: (a) a medical history that prevented sports practice (cardiovascular disease, severe visual impairment, neurological disease, or pulmonary disease requiring oxygen use), (b) injury affecting the osteoarthromuscular apparatus at the time of the study, (c) reading or listening comprehension problems, (d) being absent during the pre- or post-test evaluation periods, (c) less than 80% attendance at the intervention sessions for the experimental groups, (d) regular practice of physical exercise or sports for one or more days a week outside of the school context, and (e) previous experience in handball or athletics. 

### 2.2. Measures

#### 2.2.1. Executive Functions

The Neuropsychological Assessment for Executive Functions in Children (ENFEN) battery [47] was used. The individual application of this test is indicated for Spanish-speaking schoolchildren between 6 and 12 years of age, and in this study, its application lasted approximately 30–40 min per participant. This battery comprises four tests that allow us to evaluate the neuropsychological development of students using activities related to executive functions, yielding a direct score for each task, which can be used to obtain typical scores that are expressed on a scale of Standard Ten (sten) scores (M = 5.5, SD = 2.0). Each of the four parts has a training rehearsal. 

The first test is called “verbal fluency” and consists of two parts: “phonological fluency” (F1) and “semantic fluency” (F2). Phonological fluency assesses the subject’s ability to say the greatest number of words beginning with the letter “M” in 1 min, whereas the second part assesses their ability to say the greatest number of words belonging to the category of “animals” in 1 min. The direct score corresponds to the number of correct words. The second test is the “trails” test and also consists of two parts: the “gray trail” (S1) and the “color trail” (S2). Part 1 (S1) consists of putting a series of numbers together in descending order (20 to 1) on a sheet of paper as quickly as possible. Part 2 (S2) consists of putting a series of numbers together in ascending order (1–21), alternating the color of the successive numbers (yellow and pink) as quickly as possible. The time each student takes to complete each part must be recorded, and the direct score is obtained from a formula where the number of correct answers, number of errors, and time spent are entered. The third test is called the “rings task” (A) and consists of 15 subtasks in which a set of rings (which increase in number, along with the number of subtasks) must be moved from an initial position to a final position using specific materials (a board with colored rings) to replicate a drawing found in the stimulus book. The time spent by the student on each subtask is recorded and the direct score is the sum of the times of all subtasks in seconds. The fourth and final test is called the “interference” test (IN) (inspired by the Stroop effect), where students must identify the color of the ink that has been used to print 39 words (color names), divided into three columns. The name of the color and the ink color in which each word is printed never match. The student must name the color of the ink for each word. The total time taken to complete the test, the number of successes, and the number of errors are recorded, which are then incorporated into a formula to obtain the direct score of the test. 

The ENFEN battery has been widely used for clinical and research purposes [48,49,50,51,52].

#### 2.2.2. Physical Activity

The physical activity of the children was evaluated using the Physical Activity Questionnaire for older Children (PAQ-C), a Spanish-validated version [53], which was designed to be applied to primary school children, approximately between 8 and 14 years of age. This questionnaire assesses the practice of physical activity that has been performed in the last 7 days through nine questions about its type and frequency. It also consists of a 10th item that is used to identify students who undertook an uncommon activity during the previous week, without them receiving a score. Responses are rated on a 5-point scale.

#### 2.2.3. Anthropometric Parameters and Physical Fitness

Body height was measured without shoes, with an accuracy of 0.1 mm and a range of 0–2.50 m, using a portable stadiometer (Seca & Co., KG, Hamburg, Germany). Body weight (kg) was evaluated wearing a T-shirt, shorts, and no shoes, using a digital scale (TANITA^TM^, model 331, Tokyo, Japan). These anthropometric variables were measured according to the procedure described by Ross and Marfell-Jones [54]. The body mass index (BMI) was calculated as body mass divided by the square of the body height (kg/m^2^). In addition, waist circumference (WC; cm) was measured over the rim of the iliac crest through the umbilicus, using a flexible measuring tape, with an accuracy of 0.1 cm. The waist-to-height ratio (WtHR) was obtained by dividing the WC by height. A WtHR value of >0.5 is considered a cardiometabolic risk factor according to international standards [55]. 

To evaluate lower body muscular power, the standing long jump test (SLJ) was used [56], in which participants jump forward as far as possible by swinging their arms, bending their knees, and taking both of their legs off the ground simultaneously. The distance is measured in centimeters (cm) from the starting line marked on the floor to the landing point, at the back of the heel. To measure sprint performance, the 10 × 5 m agility shuttle run test [56] was used, wherein participants run as fast as possible between two cones that are positioned 5 m away from each other, performing five cycles in total (50 m) [56]. The time taken by each participant to perform the five cycles is measured in seconds. Upper body muscular strength was assessed via handgrip strength (HGS) using a JAMAR (brand) hydraulic dynamometer (Hydraulic Hand Dynamometer^®^ Model PC-5030 J1, Fred Sammons, Inc., Burr Ridge, IL: USA), with an accuracy of 0.1 lbf, where participants had to grasp the handgrip dynamometer with their dominant hand (with their arm hanging next to the body) and squeeze as hard as possible for at least 2 s. The best of two attempts was considered [56]. The six-minute walk test (SMWT) is a submaximal exercise test that was used to evaluate aerobic fitness [57,58,59], where participants were asked to walk as fast as possible, without running, in a 30-m-long corridor that was marked at each end. The distance traveled in meters after 6 min was recorded.

### 2.3. Overall Procedure

In the first instance, the characteristics and scope of the study were discussed with the school’s management and faculty, requiring authorization to incorporate participants beginning enrollment in formal sport-based curricular activities (i.e., 5th and 6th primary grades) [60,61]. Once authorization was obtained from the school, the same information was relayed to the parents or legal guardians of the selected classes by means of written communication, and an Informed Consent was sent out. In this document, parents were asked if their child presented with any of the exclusion criteria included as part of the research, indicating that in such cases they could not be included in the study. In addition, they were asked if they wished for their child to participate in an extracurricular sports workshop in that year and were informed that, if they reported their decision as positive, the allocation would be randomly assigned between two options, handball or athletics. In the event that parents and students wanted to participate in the study without playing sports, it was indicated that they would be included in the control group and receive the corresponding evaluations. Next, the children whose participation was authorized were informed about the characteristics of the study, and their Informed Assent was requested. Legal guardians’ and students’ preferences determined their enrollment (or lack of enrollment) in sport-specific activities, considering motivation and positive experiences as key elements of the learning process and cognitive outcomes [49]. 

Informed consent was sent to 140 parents (i.e., one for each student), distributed across four courses with 35 students each. Signed documents were returned (*n* = 127), and students’ assent was obtained from 117 participants. Thirty-three students decided not to be involved in the sport-specific training groups, although one student changed address, and two students did not attend the measurement sessions, resulting in the exclusion of these three participants from the study. From the 84 students that decided to practice a sport, 24 were excluded due to exclusion criteria during the intervention period. 

Once the Informed Consents and Assents were signed, the PAQ-C was administered to all participants by means of a direct interview. In this manner, through information provided by the children themselves, it was verified that they did not engage in physical exercise or sports outside of the school context in addition to physical education classes. The boys and girls who met all the criteria to join the study were randomly assigned to the experimental or control groups, depending on their decision to practice or not to practice sports.

Each evaluation period (pre- and post-test) included measurements with the reported instruments in two sessions during the school day. Anthropometric parameters (weight, height, BMI, WC, and WtHR) were evaluated by the school’s nurse, who had more than 10 years of professional experience and was trained by the research team to perform these measurements. The tests to measure physical fitness (SLJ, 10 × 5 m sprint, HGS, and SMWT) were performed by the school’s team of physical education teachers, who also received prior training. The evaluation of physical activity by means of the PAQ-C questionnaire and the evaluation of executive function with the ENFEN battery were performed by a team of speech therapists who were specially trained for the direct and individual application of these instruments in an individual room for each participant, free of distractions and always in the same order, ensuring that the application conditions were the same for all students. No evaluator was previously aware of the group to which each student was assigned.

The first experimental group had the handball intervention plan applied; the second experimental group had the athletics intervention plan applied, which only included track events; and the control group did not participate in sports activities. All groups took part in physical education classes for 180 min per week (i.e., two 90-min sessions) as part of their regular school activities.

### 2.4. Intervention Characteristics

The children who participated in extracurricular workshops had an additional 120 min per week of the sport to which they were assigned in two weekly sessions of equal duration (i.e., 60 min) and on days other than the physical education days. This implies that participants in both experimental groups practiced structured physical activity for 4 days a week (2 days of physical education and 2 days of extracurricular sports). The workshops were held at the same school after the end of regular classes and were led by two physical education teachers from the same school. The intervention had a total effective duration of 12 weeks, not including the initial and final evaluation periods. A perceived exertion scale for children (EPInfant scale) [62,63] was administered to participants immediately after the end of each handball and athletics training session. This instrument was administered by the physical education teachers in charge using numerical and verbal descriptors and illustrations. The perceived exertion range was between 5 and 7 per training session according to the EPInfant scale, which corresponds to moderate to vigorous intensity [62,63].

The handball training method incorporated blocks of tasks, exercises, or games, combining the phases of attack, retreat, defense, and counterattack in an analytical or combined manner. Likewise, variables such as game space, number of players and resolution time were also manipulated. The structure of the program consisted of 10 min of basic sports training, 40 min of the main exercise, and 10 min of cooling down with stretching. The basic sports training included progressive movements, joint mobility, and pair work (e.g., jumping over a person). The main exercise involved 5 min of individual improvement tasks (e.g., 1 × 1 throw), 10 min of one-phase improvement tasks (e.g., 3 × 2 in three areas of the attack field), and 10 min of phase combination tasks (e.g., 3 × 3 counterattack–retreat), starting with a time-limited defensive phase to solve the task and 15 min of real play (e.g., matches combining equality and numerical inequality), looking for variability in the task. The final 10 min included cooling down and stretching. The handball training method is shown in Table 1.

The athletic training method consisted of 10 min of basic sports training, 40 min of the main exercise, and 10 min of cooling down with stretching. The basic sports training included 5 min of continuous aerobic jogging plus 5 min of joint mobility and stretching. The main exercise involved 10 min of basic athletic abilities (e.g., running in place, skipping, alternating jumps) with a technical component, 10 min of lifts and repetitions through the set, 10 min of introduction to fartlek (i.e., similar to high-intensity interval training), and 10 min of continuous running. The final 10 min included cooling down and stretching (Table 2).

### 2.5. Statistical Analysis

The statistical analysis was performed using STATA v15.0 software. Descriptive data are presented as the mean and standard deviation. A *t*-test analysis was used to compare differences in anthropometric characteristics, physical activity, physical fitness, and sten scores within-groups. For between-group comparisons, the proportions between categorical variables according to changes in executive function scores were compared using a Chi-squared test. Pearson correlation coefficients were calculated to examine associations between PAQ-C scores and delta (Δ) score changes pre-intervention and post-intervention in sten scores for ENFEN tasks. The variables associated with loss and null post-intervention with the change in ENFEN tasks were assessed using a logistic regression. *p* values of <0.05 were considered statistically significant. 

## 3. Results

### 3.1. Demographic Characteristics, Anthropometric Parameters, Physical Activity, Physical Fitness, and Executive Functions between Groups

Table 3 describes the pre- and post-intervention characteristics of the three groups, noting that in the control group, the score for F1 and IN dimensions increased at the 12-week (*p* < 0.05) follow-up, whereas the intervention with athletics and handball for 12 weeks promoted an increase in PAQ-C scores (athletics, *p* = < 0. 001; handball, *p* = 0.002), SMWT (*p* = < 0.001 for both groups), 10 × 5 m sprint (handball only, *p* = 0.008), SLJ (athletics only, *p* = 0.04), and HGS (handball only, *p* = 0. 037), as well as an increase in the F2 task score (*p* = 0.007) in the athletics group and the IN task score (*p* = 0.034) in the handball group.

### 3.2. Frequency of Change Pre- and Post-Intervention for Executive Function Tasks between Groups

**Figure 1:**
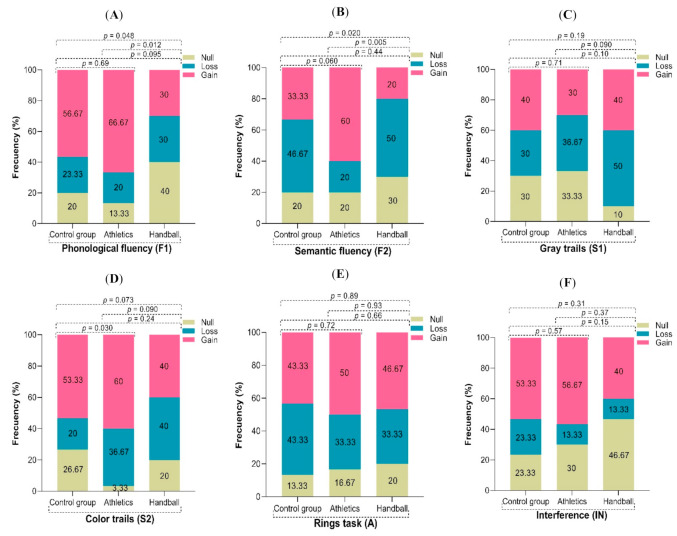
Frequency of change between pre- and post-intervention time points for sten scores on ENFEN tasks between groups. (**A**) Phonological fluency; (**B**) Semantic fluency; (**C**) Gray trails; (**D**) Color trails; (**E**) Rings; (**F**) Interference. ENFEN = Neuropsychological Assessment for Executive Function in Children battery.

In order to determine the proportion of children who showed a positive change (gain) or a decrease (loss) or who remained unchanged (null) between pre-intervention and post-intervention time points, we calculated the change (Δ) between the two observations for each executive function task between the groups. We observed that the proportion of children with gains in the F1 (Figure 1A) and F2 (Figure 1B) components was higher among the children with the athletics intervention (*p* = 0.012 and *p* = 0.005) compared with the handball group, whereas the gains for the S2 component in the athletics group showed a greater change (*p* = 0.030) in comparison with the control group (Figure 1D). 

### 3.3. Physical Activity and Executive Function

**Figure 2:**
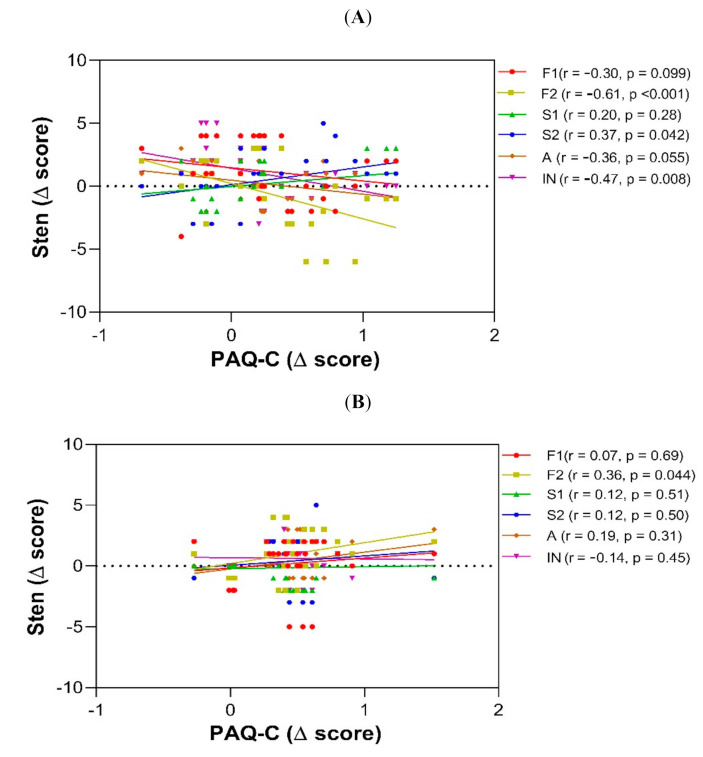

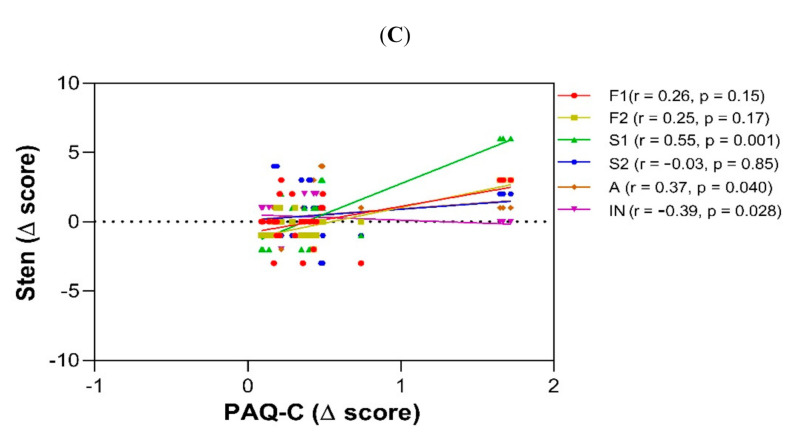
Correlation analysis between pre- and post-intervention differences in PAQ-C scores and ENFEN sten scores. (**A**) Control group; (**B**) Athletics; (**C**) Handball. Spearman correlation (rho, correlation coefficient). ENFEN = Neuropsychological Assessment for Executive Function in Children battery.

To assess the relationship between physical activity according to the PAQ-C score and ENFEN tasks, we performed a correlation between the Δ of change in the PAQ-C score derived from the pre- and post-intervention comparisons and the Δ of change between the pre- and post-interventions of each ENFEN task in each study group. Although there were children in the control group whose physical activity increased, this increase was positively related to the S2 component (r = 0.37, *p* = 0.042) but showed a negative relationship with the F2 (r = −0.61, *p* <0.001) and IN components (r = −0.47, *p* <0.008) (Figure 2A). In contrast, in the athletics group, the change in the PAQ-C score was positively related to the F2 sten score (r = 0.36, *p* = 0.044) (Figure 2B). In the group of boys in the handball group, the greater was the positive change in the PAQ-C score, the higher were the S1 (r = 0.55, *p* = 0.001) and A scores (r = 0.37, *p* = 0.04), although the IN score was lower (r = −0.39, *p* = 0.028) (Figure 2C). 

**Figure 3:**
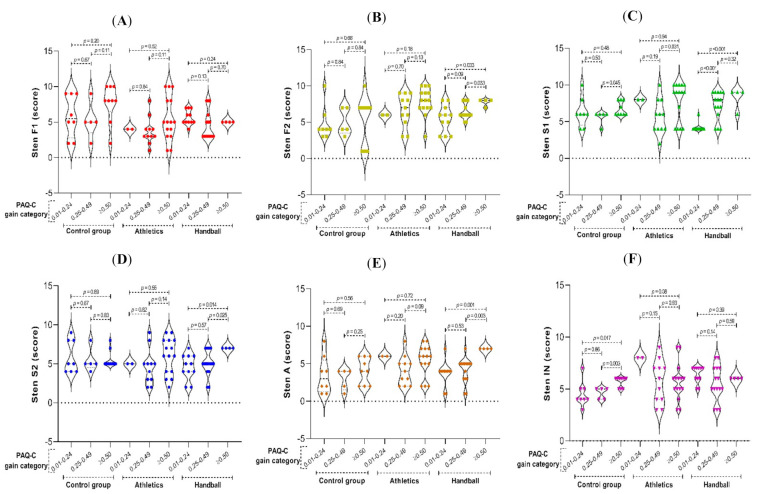
Sten score gain associations by study group. (**A**) F1; (**B**) F2; (**C**) S1; (**D**) S2; (**E**) A; (**F**) IN. Comparison of means (± standard deviation) by t-student.

Moreover, we categorized the post-intervention PAQ-C score gain and assessed the association with the ENFEN sten scores (Figure 3). A gain of ≥0.50 PAQ-C points was found to be associated with a significant increase in F2 (*p* = 0.03), S1 (*p* < 0.001), S2 (*p* = 0.014), and A (*p* = 0.001) sten scores in the handball group. For the athletics group, a gain of ≥0.50 PAQ-C points was associated with a significant increase in the S1 sten score (*p* = 0.031). For the control group, a gain of ≥0.50 PAQ-C points was associated with a significant increase in the S1 (*p* = 0.045) and IN (*p* = 0.003) sten scores. 

### 3.4. Factors Related to a Loss or Null Change in Executive Functions

Finally, we investigated the factors that were related to a loss or null change in ENFEN sten scores and identified that being male (OR = 2.7 (1.15–6.36), 0.02) or having presented with low HGS (OR = 4. 0 (1.64–10.0), 0.002) or WtHR > 0.5 (OR = 4.82 (1.26–18.36), 0.02) scores prior to the intervention were factors associated with a loss or null change of the sten score A post-sports intervention. On the other hand, having a low HGS score was shown to be a risk factor for a loss or null change in the IN sten score (OR = 3.23 (1.31–7.92), 0.01), and WtHR > 0.5 represented a variable associated with a loss or null change in F2 (OR = 12.1 (1.51–96.2), 0.019) (Table 4).

## 4. Discussion

This study aimed to analyze the differences in different executive function dimensions in children practicing an open-skill sport (handball) and a closed-skill sport (athletics) and controls that did not participate in sports activities after a 12-week intervention period. 

### 4.1. Changes Pre- and Post-Intervention for Executive Function Tasks between Groups

Our findings indicate that the handball group evidenced an increase in the ENFEN IN task, reflecting an improvement in the inhibitory control executive function [64,65,66]. The athletics group, on the other hand, increased their F2 task performance, which has been described as a measure of cognitive flexibility [64,67,68] and as an indirect measure of working memory [52,69]. The results of our study are comparable to those of the study conducted by Cho et al. [70], who analyzed changes in executive function in a sample of elementary school students after a 12-week intervention period with another open-skill sport (taekwondo), finding an improvement in inhibitory control. Our results are also consistent with those of Formenti et al. [41], who found significantly higher inhibitory control performances in children practicing open-skill sports in comparison with those practicing closed-skill sports. In contrast, Heppe et al. [71] evidenced better motor inhibitory control, with expert handball players demonstrating remarkably shorter response times compared with recreational handball players and physically active controls. Other studies that evaluated executive functions after intervention programs with open-skill sports (team sports) did not find notable changes in inhibition ability, which could be related to the shorter duration of their programs (6–8 weeks) [29,33,45] or methodological limitations associated with the measurement of executive functions [42]. In the case of athletics, Venckunas et al. [44] provide a precedent, having investigated the effect of a 7-week interval running program in a sample of adolescents, demonstrating significant improvements in cognitive flexibility and aerobic fitness after the intervention period. A cross-sectional study by Takahashi and Grove [72] compared the acute effect of badminton versus running on inhibitory control in young adults using the Stroop test, evidencing a remarkably better performance in the badminton group. Although the acute effect of exercise is not equivalent to its chronic effect, this constitutes additional background information that is associated with the absence of an inhibition change in our study in the athletics group.

The control group also showed performance improvements after the 12-week follow-up for the F1 and IN tasks, reflecting improvements in cognitive flexibility, working memory, and inhibitory control. This increase, although not corresponding to our initial hypothesis, may be related to the specific moment when data were collected, precisely when students were returning to the in-person school context after an extensive period of restrictions on mobility and free movement during the COVID-19 pandemic, which resulted in schools being kept closed for more than 18 months in Chile. Although neither physical activity nor physical condition showed significant changes in the control group, it is inferred that the positive stimuli typical of the return to in-person schooling (social, emotional, and cognitive) may have stimulated the executive functions of these children, because they were forced to respond to the increased demands of the school context [73,74]. 

### 4.2. Frequency of Change Pre- and Post-Intervention and Factors Related to a Loss or Null Change in Executive Functions

When comparing the proportion of children who showed a positive change or gain in executive function in both groups who played sports, the athletics group was found to significantly outperform the handball group in both verbal fluency tasks (F1 and F2), indicating that a higher percentage of children in this group experienced an improvement in their cognitive flexibility and working memory performances. The athletics group also significantly outperformed the control group by the same proportion on the color trails task, which assesses cognitive flexibility. However, for the executive function tasks, there was a percentage of participants who showed null or loss changes, which were significantly related to being male (S1 and A), having presented with a low HGS (A and IN), or a WtHR value of >0.5 (F2 and A) prior to the intervention. Mora et al. [75] previously reported a significant positive association between HGS and planning ability, whereas Veraksa et al. [76] demonstrated a significant positive relationship between throwing and inhibition tests, with both studies being performed in children. Flores et al. [77] also provided background data associating executive functions in children and adolescents with strong predictors of cardiovascular disease, including WtHR, indicating that cognitive test scores decrease as the number of cardiovascular risk factors involved increases.

### 4.3. Sports, Physical Activity, Physical Fitness, and Executive Functions

The groups that participated in sports activities showed a significant increase in physical activity (PAQ-C) and aerobic fitness (SMWT). Previous research suggests that participating in regular aerobic sports activity can result in positive changes in cognitive performance, which may have contributed to the improvements observed in executive function [31,78,79]. Other recent studies have also demonstrated that a higher level of physical activity is associated with greater executive function development in children [80,81]. The handball group showed additional significant improvements in sprint performance (10 × 5 m sprint) and upper body muscular strength (HGS), whereas the athletics group demonstrated significant improvements in lower body muscular power (SLJ). This reflects the common and differentiating characteristics of the training programs of each sport, which share an aerobic component but emphasize different dimensions of physical fitness, such as agility, speed, and strength [29,44,45]. 

Studies that have analyzed the relationship between specific physical fitness tests and executive functions in children and adolescents show considerable positive associations between upper body muscular strength (hand grip and throwing tests) and planning skills, working memory, and inhibition; between speed-agility and cognitive flexibility, inhibition, and working memory [75,76]; and between cardiorespiratory fitness and cognitive flexibility, working memory, inhibitory control, planning, and problem solving [1,37,75,82,83,84,85,86]. Studies that have merged the results of physical tests to obtain an overall measure of physical fitness identified that their improvement is associated with higher performances in cognitive flexibility [75], working memory [87], and inhibition [88]. 

A detailed analysis of the relationship between gains in physical activity and improvements in executive function tasks showed that increases in physical activity in the handball group correlated significantly with increases in attention (S1 task) and planning (A task). Furthermore, a gain of ≥0.50 points in physical activity (PAQ-C) for that group was associated with a significant increase in cognitive flexibility, working memory, attention, and planning (F2, S1, S2, and A tasks). In the athletics group, a positive change in physical activity was associated with higher cognitive flexibility and working memory (F2 task) performances, whereas an increase of ≥0.50 points in physical activity (PAQ-C) was associated with better performance in the attention (S1) task. The gain in physical activity was only related to planning ability in the handball group; furthermore, the relationship between increased physical activity and executive function improvement extended to a greater number of executive function tasks in the handball group compared with the athletics group. These findings support the idea that qualitative differences in physical activity, and specifically in sport, are associated with gains in different executive function dimensions, depending on the inherent characteristics of each discipline [11,35,40]. Within the control group, increased physical activity correlated positively with cognitive flexibility performance (S2 task), and PAQ-C score gains of ≥0.50 points were associated with improvements in attention and inhibitory control (S1 and IN tasks).

These data show differences in the improvement profile of the different executive function dimensions for an open-skill and a closed-skill sport, and evidence that the increase in physical activity above the threshold of 0.50 points in PAQ-C scores is relevant to improved performance in some executive function tasks, especially when it incorporates sports practice. In addition, working memory, cognitive flexibility, and planning appear to be more sensitive to increased sport-based physical activity, whereas inhibitory control appears to improve in response to increased physical activity by ≥0.50 points in PAQ-C scores, even in the absence of sports practice, given the incorporation of stimuli associated with the academic environment, which was observed for the control group.

### 4.4. Advantages, Limitations, and Future Lines of Research

This study has the advantage of thoroughly describing the specific contributions that regular handball and athletics practice have on executive functions, covering its three main dimensions and planning ability, all of which are highly relevant in the school context and physical education class design. In addition, it provides information on the relationships between physical activity and executive function tasks, allowing for the differentiation of the conditions that involve sports practice, while analyzing the variables involved with the absence of change in executive function after the intervention period. A limitation of this study is that the sample was obtained in a private school located in an urban area, which limits the generalization of the results to the socioeconomic strata in which the children develop. In addition, the battery of tests used to evaluate executive function does not allow for the isolation of the working memory dimension with a specific task, which could be incorporated into future research. It is suggested that the specific contribution of different sports modalities to executive functions continues to be analyzed through longitudinal studies in schoolchildren, using tasks that allow for a clear view of the effect on each modality of executive function to be obtained. This could favor the generation of strategies that enhance children’s cognitive development and academic success at school age.

## 5. Conclusions

Schoolchildren who practice sport in a sustained manner for 12 weeks demonstrate improvements in their performance of executive functions in cognitive flexibility, working memory, and inhibitory control, and the increase in physical activity in these groups is also associated with improvements in attention and planning, primarily in the group that practices a team sport. 

The present findings suggest the need to implement sports interventions that favor increased physical activity and physical fitness in schoolchildren to enhance their cognitive development.

## Figures and Tables

**Table 1:** Handball training program.

Procedure	Contents	Time (min)
Basic sports training	Progressive movements, joint mobility, and work in pairs	10
Main exercise	Individual improvement tasks. Example: 1 × 1 throw	5
	One-phase improvement tasks. Example: 3 × 2 in three areas of the attack field	10
	Phase combination tasks. Example: 3 × 3 counterattack–retreat	10
	Real play. Example: Matches combining equality and numerical inequality	15
Cooling down	Stretching	10

**Table 2:** Athletics training program.

Procedure	Contents	Time (min)
Basic sports training	Continuous aerobic jogging	5
	Joint mobility and stretching	5
Main exercise	Basic athletic abilities with a technical component	10
	Lifts and repetitions through the set	10
	Introduction to fartlek	10
	Continuous run	10
Cooling down	Stretching	10

**Table 3:** Demographic characteristics, anthropometric parameters, physical fitness, and ENFEN tasks by children according to the sports practice intervention.

Children’s Characteristics	Pre-Intervention	Post-Intervention	*p*-Value	Pre-Intervention	Post-Intervention	*p*-Value	Pre-Intervention	Post-Intervention	*p*-Value
Groups	Control Group			Athletics			Handball		
Age	11.45 ± 0.60	-	-	11.4 ± 0.85	-		11.41 ± 0.59	-	0.93
Sex			-			-			-
Girls	15 (50%)	15 (50%)		14 (46.7)	14 (46.7)		15 (50.0)	14 (46.7)	
Boys	15 (50%)	15 (50%)		16 (53.3)	16 (53.3)		15 (50.0)	16 (53.3)	
Anthropometric parameters									
Weight (kg)	42.7 ± 7.67	43.1 ± 7.7	0.87	44.5 ± 7.65	45.28 ± 8.16	0.69	43.6 ± 8.17	43.86 ± 8.29	0.90
Size (m)	1.48 ± 0.05	1.50 ± 0.05	0.27	1.52 ± 0.048	1.53 ± 0.049	0.31	1.49 ± 0.08	1.51 ± 0.08	0.52
WC (cm)	70.1 ± 10.7	69.8 ± 10.34	0.90	71.46 ± 5.78	70.3 ± 5.16	0.43	70.4 ± 6.44	69.86 ± 6.26	0.73
WtHR (WC/height)	0.47 ± 0.07	0.46 ± 0.07	0.72	0.46 ± 0.04	0.45 ± 0.038	0.27	0.47 ± 0.04	0.46 ± 0.03	0.43
BMI (kg/m^2^)	19.3 ± 3.7	19.1 ± 3.4	0.78	19.17 ± 3.1	19.2 ± 3.34	0.97	19.26 ± 2.44	19.0 ± 2.41	0.67
Physical fitness									
PAQ-C	2.73 ± 0.96	3.02 ± 1.0	0.26	2.6 ± 0.56	3.09 ± 0.51	<0.001	2.49 ± 0.58	3.4 ± 0.54	0.002
SMWT (m)	381.4 ± 98.5	381.5 ± 95.2	0.91	384.9 ± 51.5	726.5 ± 70.6	<0.001	330.3 ± 16.9	734.0 ± 74.6	<0.001
10 × 5 m sprint (s)	23.4 ± 2.7	23.7 ± 2.7	0.63	22.49 ± 2.32	21.58 ± 1.72	0.08	21.94 ± 2.01	20.6 ± 1.75	0.008
SLJ (cm)	134.7 ± 15.3	134.4 ± 14.4	0.93	143.8 ± 21.7	155.6 ± 23.1	0.04	152.7 ± 12.1	157.26 ± 14.56	0.19
HGS (kg)	14.7 ± 3.3	14.9 ± 3.25	0.81	17.86 ± 7.03	21.0 ± 5.93	0.06	18.96 ± 4.12	21.16 ± 3.86	0.037
ENFEN tasks									
Phonological fluency (F1)	11.96 ± 4.7	14.76 ± 4.49	0.021	10.5 ± 3.92	11.73 ± 4.83	0.28	11.93 ± 3.7	13.03 ± 3.15	0.22
Sten F1	5.03 ± 2.8	6.2 ± 2.56	0.098	4.43 ± 2.34	4.66 ± 2.55	0.71	4.96 ± 2.37	5.03 ± 1.6	0.89
Semantic fluency (F2)	18.16 ± 5.7	17.43 ± 4.73	0.59	18.7 ± 2.7	21.8 ± 5.54	0.007	19.7 ± 3.83	19.8 ± 4.21	0.92
Sten F2	5.83 ± 2.76	5.23 ± 2.34	0.36	6.2 ± 0.96	7.23 ± 1.97	0.012	6.46 ± 1.47	6.26 ± 1.52	0.60
Gray trails (S1)	29.7 ± 8.66	32.6 ± 10.6	0.26	36.97 ± 12.64	37.84 ± 16.6	0.82	29.9 ± 10.76	32.34 ± 10.91	0.38
Sten S1	6.03 ± 2.05	6.26 ± 1.38	0.60	7.13 ± 2.1	7.0 ± 2.49	0.82	5.86 ± 2.3	6.23 ± 2.17	0.52
Color trails (S2)	16.64 ± 6.63	18.4 ± 4.73	0.24	16.58 ± 4.4	18.57 ± 7.0	0.19	15.9 ± 5.5	17.2 ± 3.95	0.32
Sten S2	4.93 ± 2.3	5.46 ± 1.56	0.29	4.93 ± 1.76	5.36 ± 2.17	0.39	4.66 ± 1.95	5.13 ± 1.65	0.32
Rings task (A)	186.77 ± 18.28	175.0 ± 32.9	0.09	176.8 ± 33.0	171.4 ± 43.7	0.58	185.61 ± 32.6	174.5 ± 36.0	0.21
Sten A	3.93 ± 0.86	4.06 ± 1.94	0.72	4.43 ± 1.38	4.86 ± 2.08	0.34	3.86 ± 1.85	4.36 ± 1.82	0.29
Interference (IN)	74.13 ± 21.69	86.39 ± 14.3	0.012	85.0 ± 19.73	92.3 ± 21.83	0.18	84.0 ± 11.96	90.9 ± 12.49	0.034
Sten IN	4.6 ± 1.84	5.5 ± 1.4	0.038	5.3 ± 1.82	5.93 ± 1.85	0.18	5.56 ± 0.97	5.9 ± 1.32	0.27

The data shown represent the mean ± standard deviation. ENFEN = Neuropsychological Assessment for Executive Function in Children battery; BMI = body mass index; WC = waist circumference; WtHR = waist-to-height ratio; PAQ-C = Physical Activity Questionnaire for Older Children; SMWT = six-minute walk test; SLJ = standing long jump test; HGS = handgrip strength.

**Table 4:** Association of loss or null change parameters on ENFEN task scores in children.

ENFEN Sten	Sex—Male	Low HGS (<3rd)	WtHR (>0.5)
F1	1.30 (0.57–2.99), 0.52	0.93 (0.39–2.18), 0.86	0.56 (0.18–1.72), 0.31
F2	1.57 (0.66–3.7), 0.30	1.87 (0.78–4.5), 0.15	12.1 (1.51–96.2), 0.019
S1	0.18 (0.07–0.48), 0.001	0.59 (0.24–1.48), 0.26	0.69 (0.23–2.08), 0.51
S2	0.76 (0.33–1.75), 0.53	1.64 (0.69–3.9), 0.25	0.77 (0.26–2.3), 0.65
A	2.7 (1.15–6.36), 0.02	4.0 (1.64–10.0), 0.002	4.82 (1.26–18.36), 0.02
IN	2.05 (0.88–4.75), 0.09	3.23 (1.31–7.92), 0.01	0.53 (0.17–1.63), 0.27

Data shown represents the OR (95% CI). HGS = handgrip strength; WtHR = waist-to-height ratio; low HGS < 3rd represents values <18 kg.

## Data Availability

The datasets that were generated and analyzed for this study can be obtained from the corresponding author.
